# Supplementary material for: A CRISPR homing screen finds a chloroquine resistance transporter-like protein of the Plasmodium oocyst essential for mosquito transmission of malaria
Source: Nat Commun. 2025 Apr 24;16:3895. doi: 10.1038/s41467-025-59099-1 (PMC12022033; doi:10.1038/s41467-025-59099-1)
Supplement: Supplementary file 1 — Supplementary Information [file 41467_2025_59099_MOESM1_ESM.pdf]

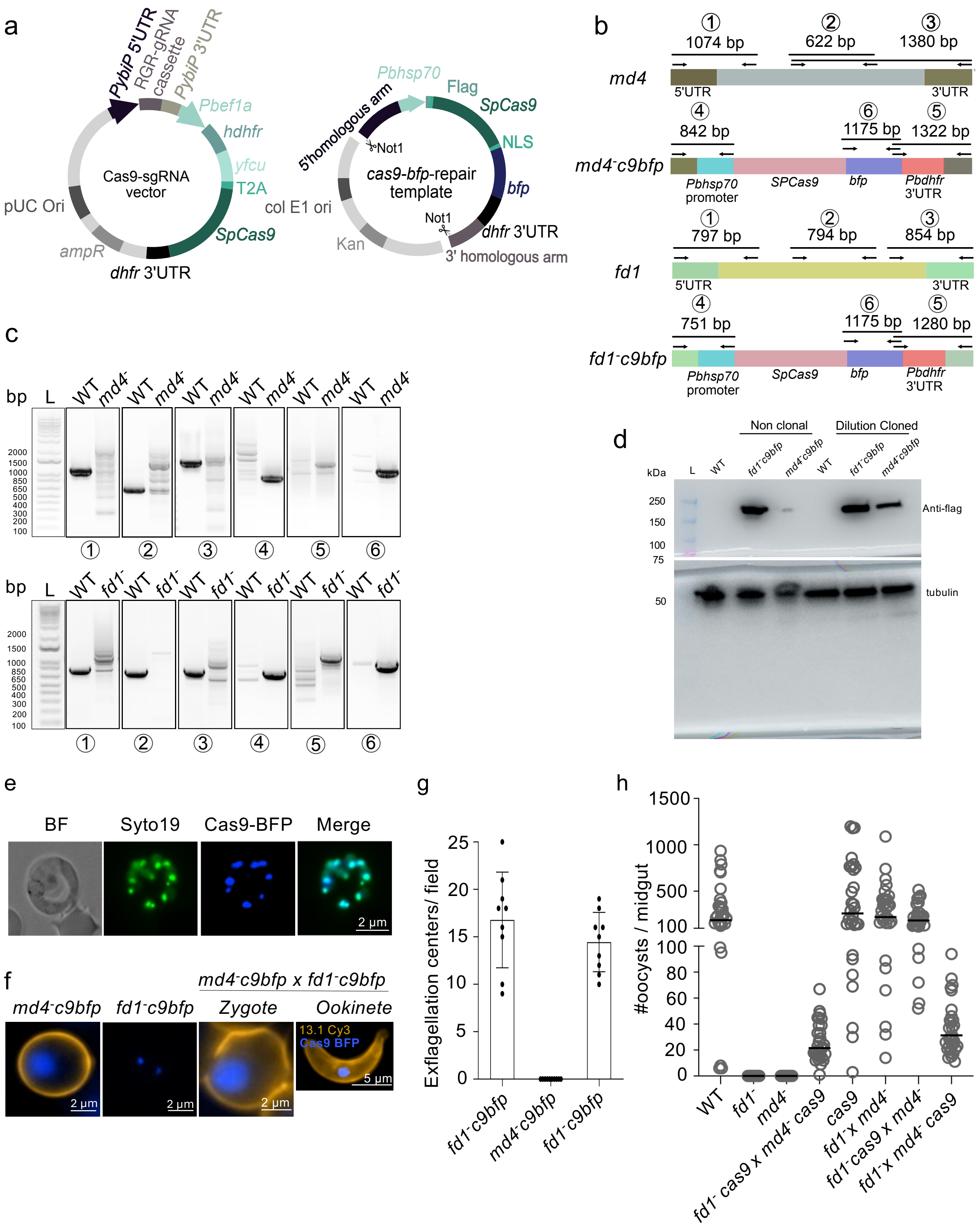

Supplementary Fig. 1: Generation of *Cas9-bfp expressing* single sex lines. **a** Schematic of vectors used to generate single sex lines. **b** Schematic showing primers used to genotype *cas9-bfp* lines. **c** Genotyping of *cas9-bfp* single sex lines. **(d, e)** Nuclear localization and expression of Cas9-BFP assessed by microscopy and western blot in mixed blood stages. The data is representative of two independent experiments **f** Expression of Cas9 BFP in activated female gametes (*md4-c9bfp*), male gametes (*fd1-c9bfp*), zygote and ookinete (*md4-c9bfp x fd1-c9bfp).* The data is representative of three independent experiments. **g** Assessment of single sex lines to form active male gametes by exflagellation assay. The data show three exflagellation experiments from each of three different infections. **h** Fitness of Cas9-expressing single sex lines determined by counting the number of oocyst in the mosquito midgut 12 days post infectious blood meal. The data are from two independent experiments with 25 infected mosquitoes in each set (n=25).

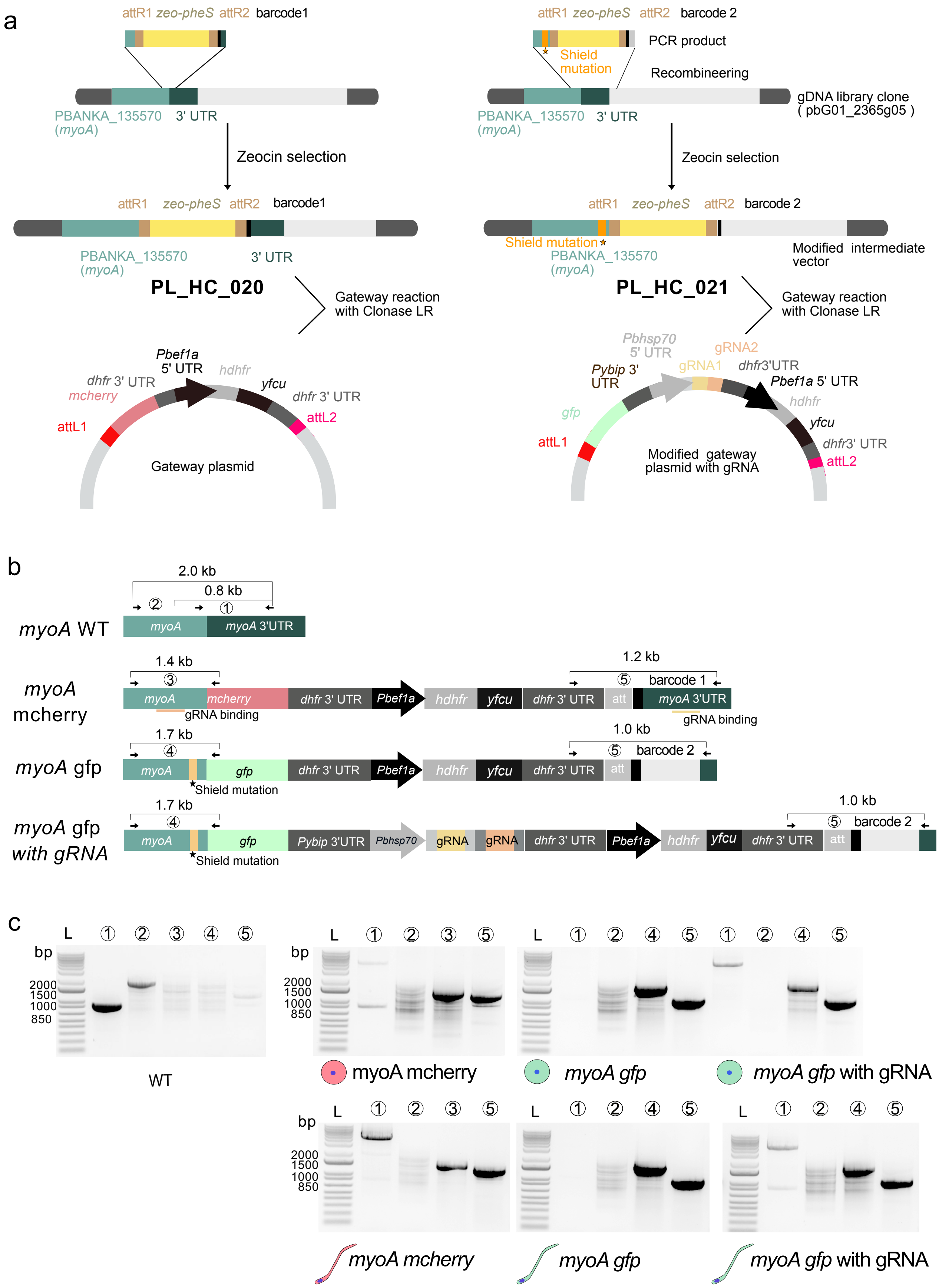

Supplementary Fig. 2: Generation of myoA mCherry and tagged parasites expressing gRNA in male (*fd1*⁻) and female (*md4*⁻) *cas9-bfp* lines.

a Schematic showing the generation of modified tagging vectors that was used to make myoA mcherry and GFP tagged parasites expressing gRNA. **b** Schematic of final tagging vector **c** Genotyping of male (*fd1*⁻) and female (*md4*⁻) -only *cas9* expressing lines transfected with the tagging vectors.

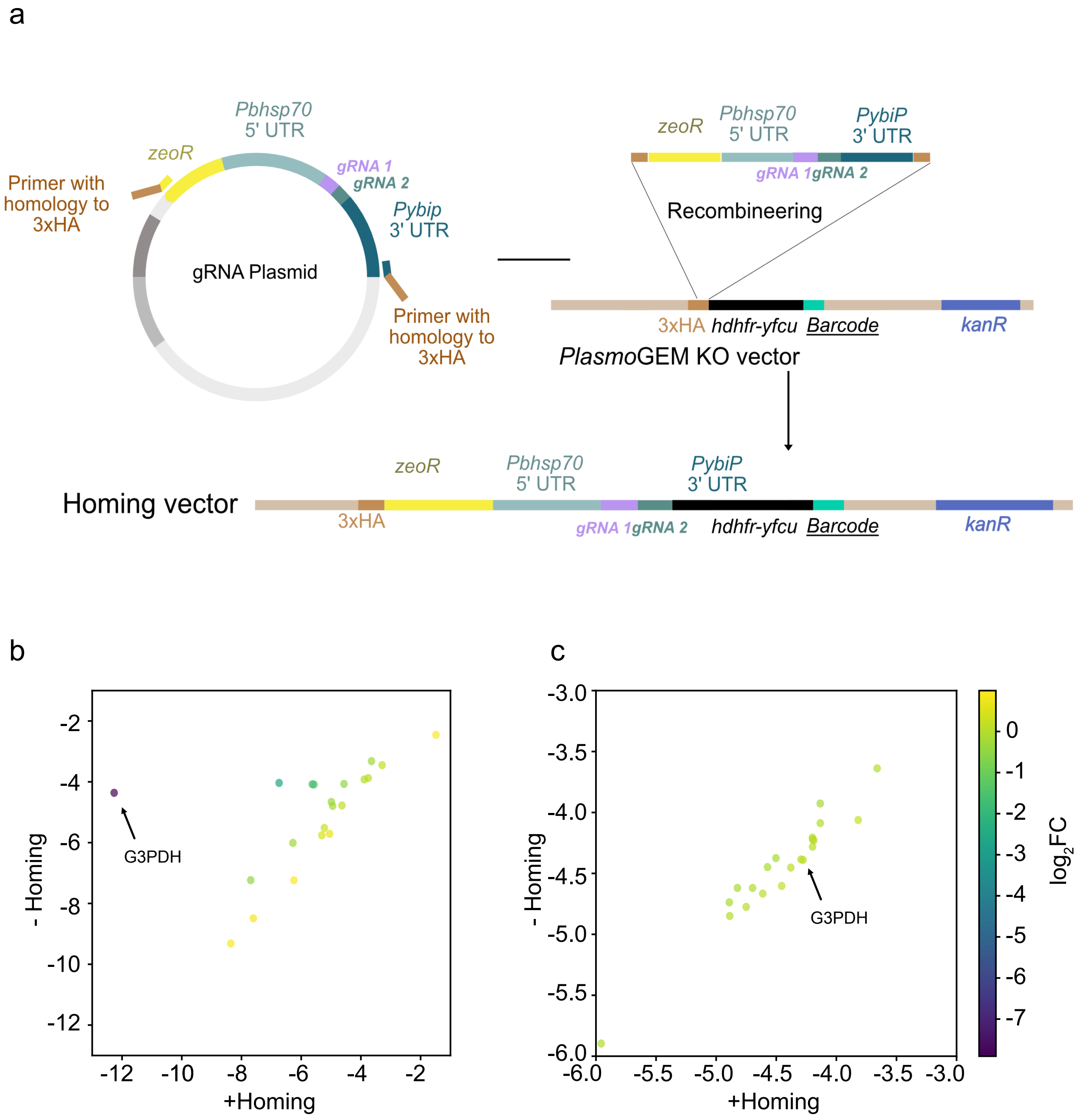

Supplementary Fig. 3: Preparation of homing-Cas9 vectors for the pilot screen and identification of possible G3PDH off-target effect.

a Schematic showing the generation of homing vectors for the pilot screen. **b** Off target score of gRNA in homing vectors visualized by a change in relative abundance of the gene in the blood stages when transfected with homing vector (+ homing) in comparison with *Plasmo*GEM vector (-homing). **c** Change in relative abundance of the homing vector in the cuvette input (+homing) in comparison with *Plasmo*GEM vector (-homing) is plotted. Different colours indicate difference in log_2-fold change between + homing in comparison to -homing.

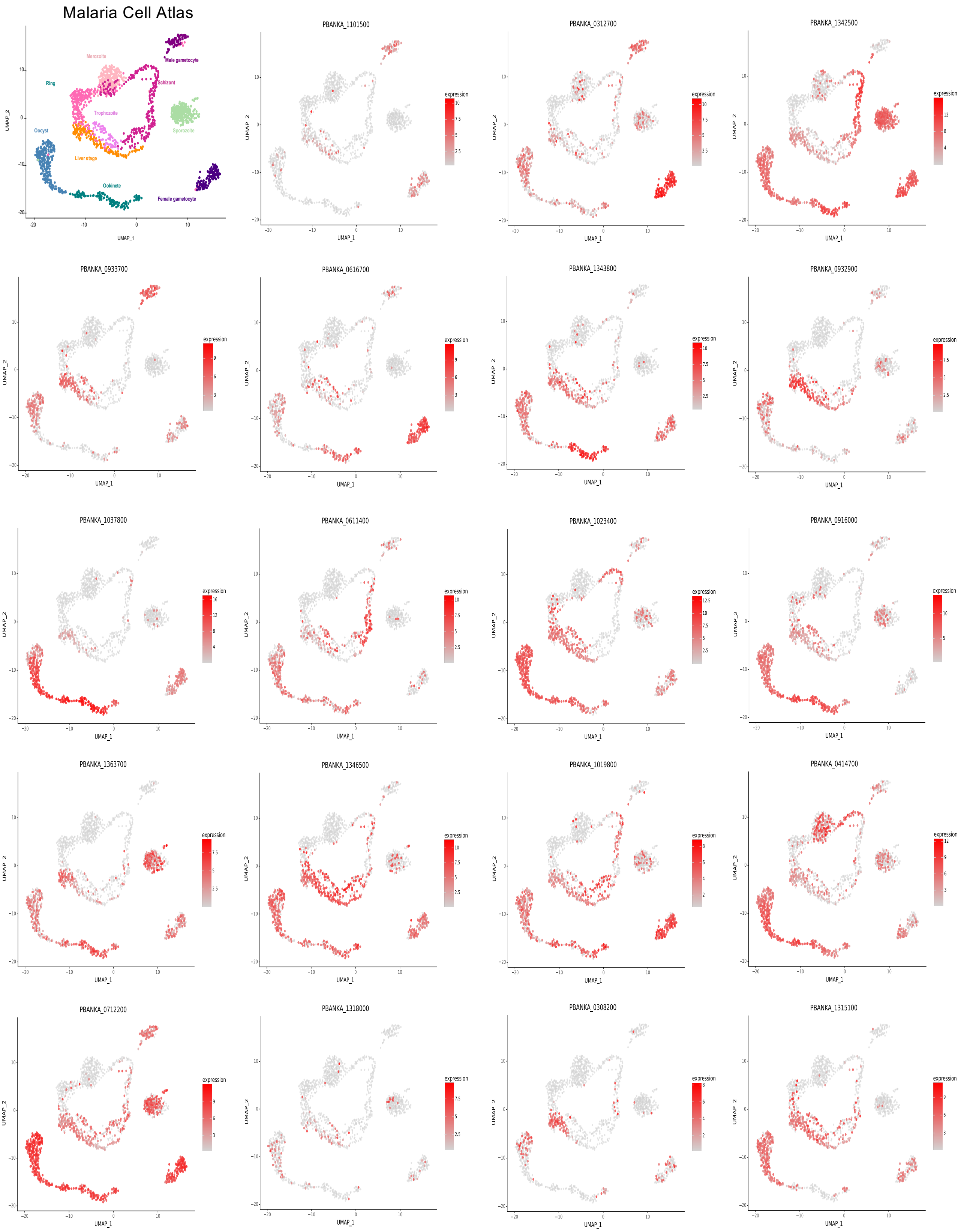

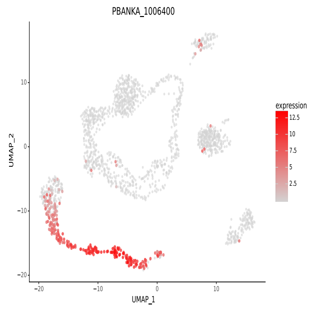

Supplementary Fig. 4: Gene expression plots.
Relative transcript abundance data from the Malaria Cell Atlas is shown for individual cells assigned to different *P. berghei* life cycle stages.

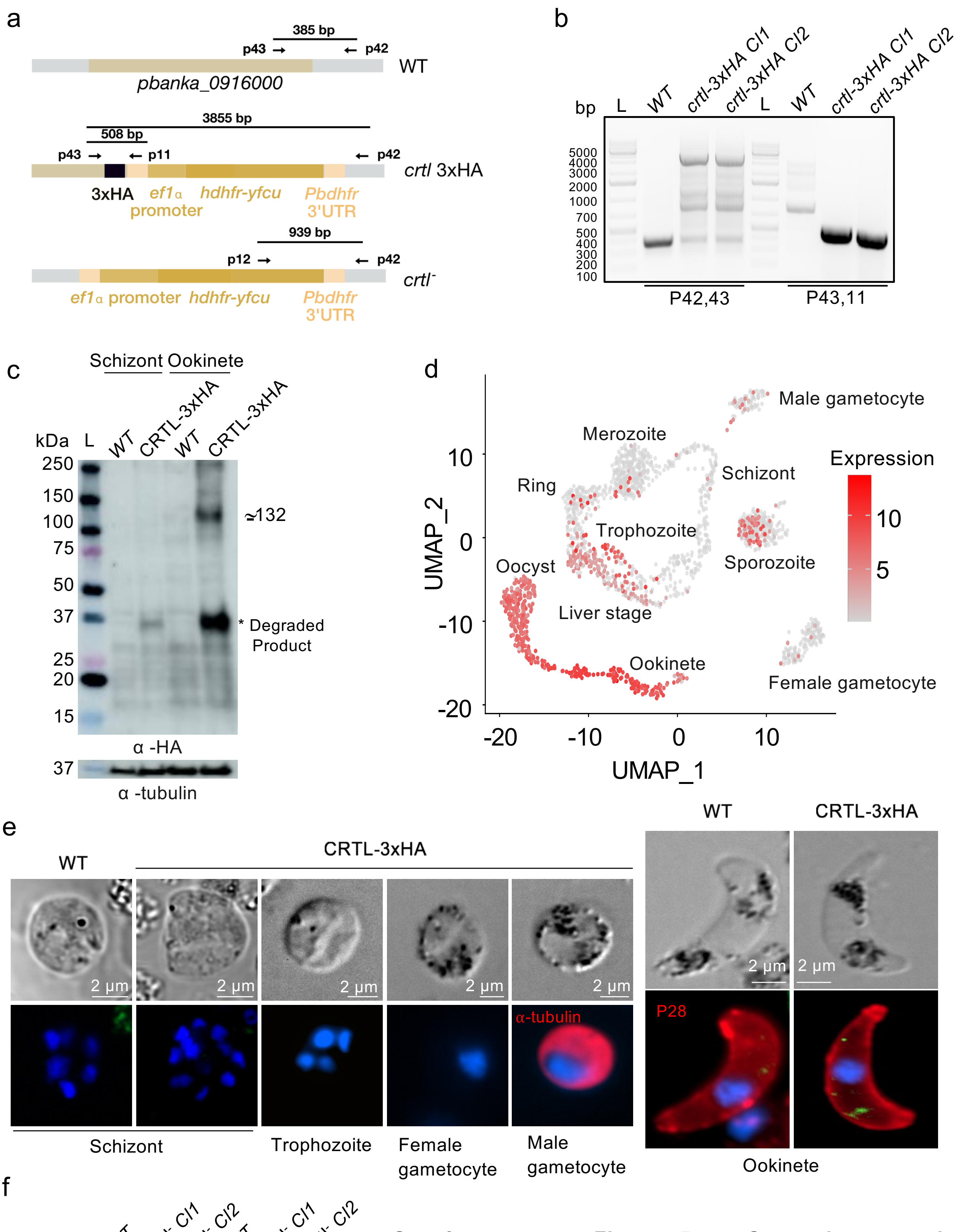

Supplementary Fig. 5: Generation and characterisation of *crtl⁻* and 3X HA tagged lines. a Schematic of the WT, disrupted and tagged *crtl* locus, showing primers that for genotyping. **b** Genotyping of *crtl* 3x-HA tagged lines. **c** Western blot analysis of CRTL-3xHA and WT parasite extracts from schizont cultures and ookinetes. **d** Stage specific transcript abundance for *crtl* from Malaria Cell Atlas data. **f** Genotyping of *crtl⁻* lines. **e Immunodetection of** CRTL-3xHA showing absence in blood stages and low expression level in ookinetes (green). Blue: DNA labelled with Hoechst; Red: P28 or α-tubulin. Images are representative of two independent experiments.

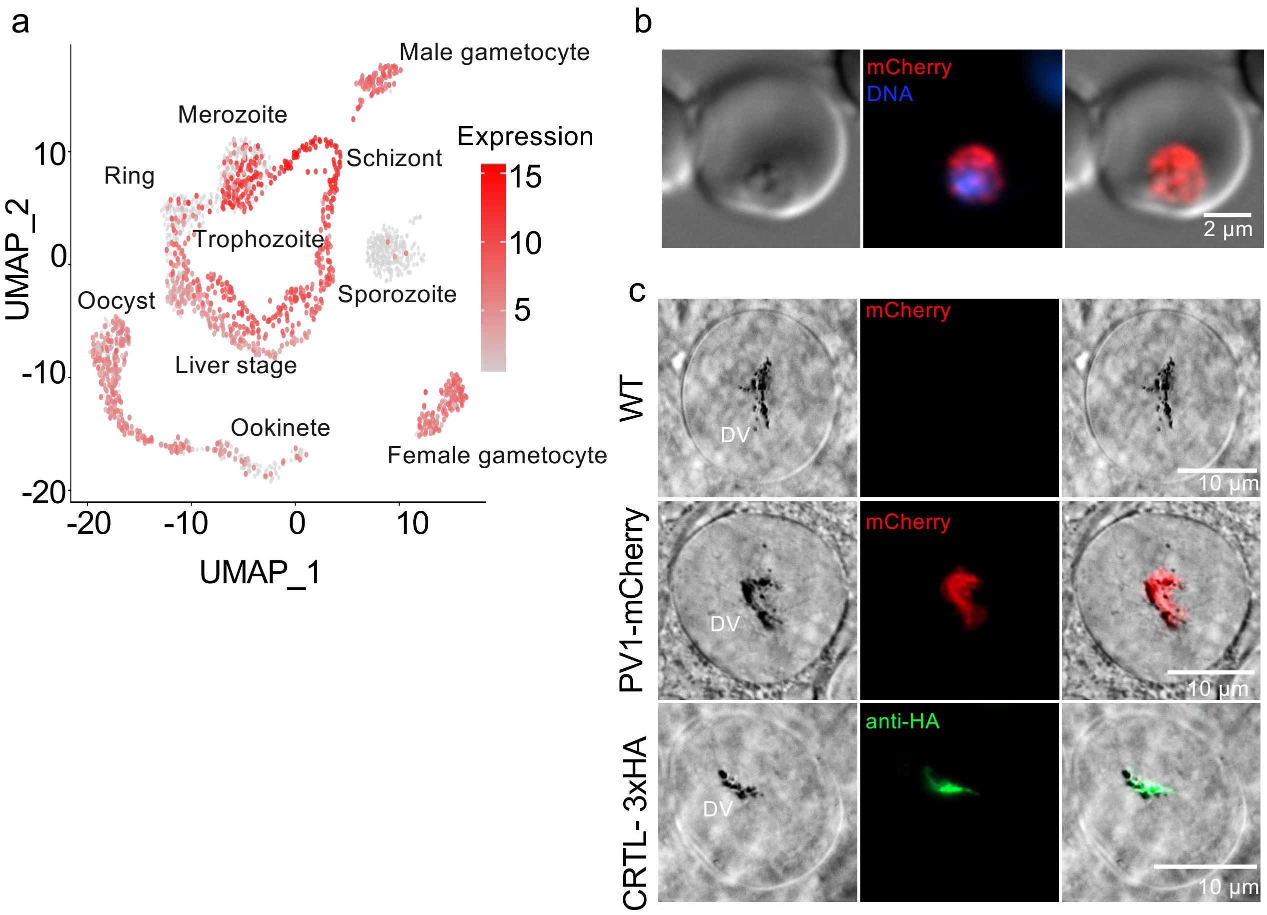

Supplementary Fig. 6 : Expression and localization of PV1.

a Single-cell transcriptomics showing expression of PBANKA_0919100 (PV1) in *P.berghei* life cycle stages. **b** Localisation and expression of PV1-mCherry in non-fixed trophozoites analysed by fluorescent microscopy. **c** Mosquitoes infected with either wild type parasites (WT), CRTL- 3xHA or PV1-mCherry were dissected on day 10 post-infection and were imaged using fluorescent microscopy. For CRTL - 3xHA infected midguts were fixed and immunostained with anti-HA antibody (green). The data representative of two separate transmission experiments, each involving the analysis of 25 infected mosquito midguts (n=25). DV - Digestive Vacuole.

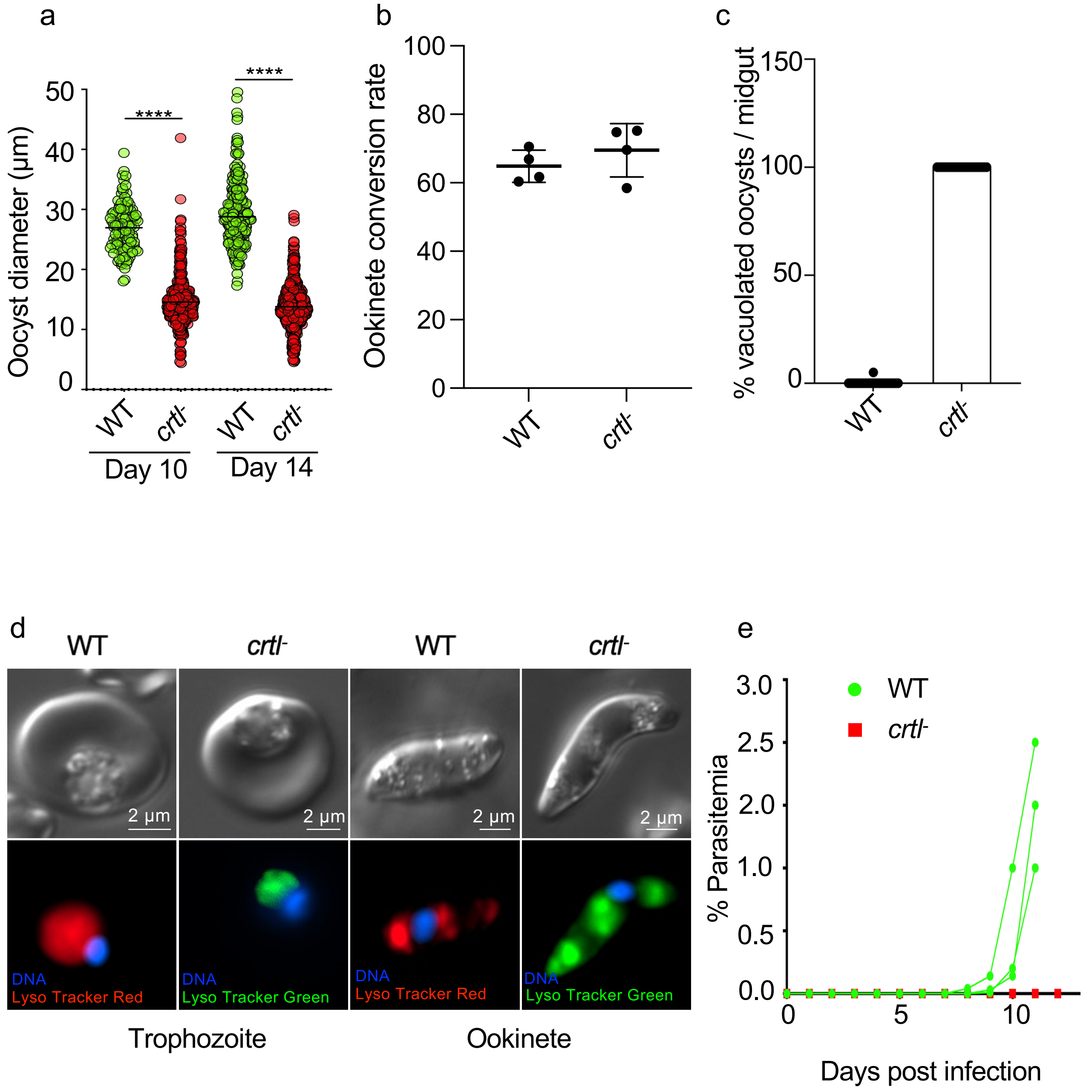

Supplementary Fig. 7: Characterisation of a *crtl* knockout clone.
a The diameter of individual *Pb Bergreen* (WT) or *Pb mCherry crtl* knock out oocysts on day 10 and day 14 post-infection is quantified and plotted. The data are from two independent experiments with 25 infected mosquitoes in each set. Oocyst diameter from randomly selected midguts were measured using ImageJ and plotted (n=200 +/- 50). (unpaired t test ****P < 0.0001, ns not significant). **b** The percentage conversion from female gametocyte to ookinete is quantified and plotted. The data is from four independent experiments. **c** Quantification of percentage vacuolated oocyst in the WT and *crtl*$^-$ day 10 infected mosquito midgut. The data is from 20 randomly selected infected mosquito midgut. **d** A representative image of *Pb Bergreen* (WT) or *Pb mCherry crtl* knock out blood stage parasites and ookinetes after staining with either Lysotracker Red (WT) or Lysotracker Green (*crtl*$^-$) to visualize acidic compartments. Hoechst was used to stain DNA. **e** Parasitaemia in mice after transmission of WT or *crtl*$^-$ parasites by infected mosquito bites (15 infected mosquitoes/ mice). Three mice were used for each infection.

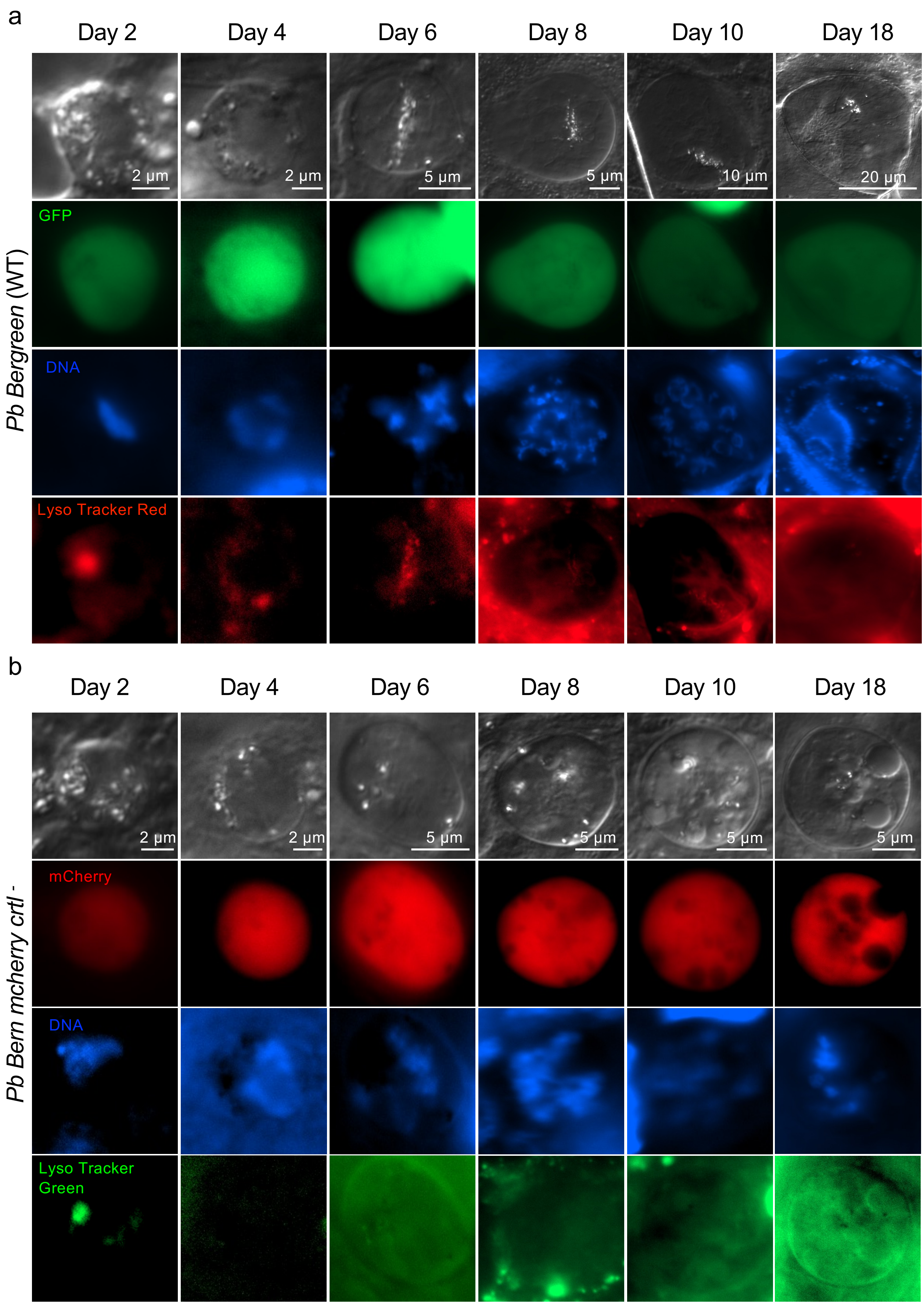

Supplementary Fig. 8: Fluorescence micrographs showing stages of oocyst development. **a** Representative images of GFP expressing *P. berghei* oocysts stained with Lysotracker Red and Hoechst for DNA. **b** Representative images of *mCherry* expressing *crtl*$^{-}$ knock out oocysts stained with Lysotracker Green and Hoechst for DNA. Images are representative of two separate transmission experiments, each involving the analysis of 15-20 infected mosquito midguts at each time point.

Supplementary References

1. Wall, R. J. *et al.* Plasmodium APC3 mediates chromosome condensation and cytokinesis during atypical mitosis in male gametogenesis. *Sci. Rep.* **8**, 5610 (2018).
2. Modrzynska, K. *et al.* A knockout screen of ApiAP2 genes reveals networks of interacting transcriptional regulators controlling the Plasmodium life cycle. *Cell Host Microbe* **21**, 11–22 (2017).
3. Tewari, R. *et al.* The systematic functional analysis of Plasmodium protein kinases identifies essential regulators of mosquito transmission. *Cell Host Microbe* **8**, 377–387 (2010).
4. Wichers-Misterek, J. S. *et al.* A microtubule-associated protein is essential for malaria parasite transmission. *MBio* **14**, e0331822 (2023).
5. Lasonder, E. *et al.* Proteomic profiling of Plasmodium sporozoite maturation identifies new proteins essential for parasite development and infectivity. *PLoS Pathog.* **4**, e1000195 (2008).
6. Lindner, S. E. *et al.* Enzymes involved in plastid-targeted phosphatidic acid synthesis are essential for Plasmodium yoelii liver-stage development. *Mol. Microbiol.* **91**, 679–693 (2014).
7. Hitz, E., Balestra, A. C., Brochet, M. & Voss, T. S. PfMAP-2 is essential for male gametogenesis in the malaria parasite Plasmodium falciparum. *Sci. Rep.* **10**, 11930 (2020).
8. Reininger, L., Garcia, M., Tomlins, A., Müller, S. & Doerig, C. The Plasmodium falciparum, Nima-related kinase Pfnek-4: a marker for asexual parasites committed to sexual differentiation. *Malar. J.* **11**, 250 (2012).
9. Tonkin, C. J. *et al.* Sir2 paralogues cooperate to regulate virulence genes and antigenic variation in Plasmodium falciparum. *PLoS Biol.* **7**, e84 (2009).
