# Supplementary material for: A CRISPR homing screen finds a chloroquine resistance transporter-like protein of the Plasmodium oocyst essential for mosquito transmission of malaria
Source: Nat Commun. 2025 Apr 24;16:3895. doi: 10.1038/s41467-025-59099-1 (PMC12022033; doi:10.1038/s41467-025-59099-1)
Supplement: Supplementary file 2 — Description Of Additional Supplementary File [file 41467_2025_59099_MOESM2_ESM.pdf]

Description of additional supplementary files

Title: Supplementary data 1 - Phenotypes and orthologs.

Description: The file explains the known functions of genes selected for the pilot screen and lists their *P. falciparum* orthologs.

Title: Supplementary Movie 1 - Time lapse of *P. berghei* WT oocyst 6 days post infection.

Description: A video of a representative *P. berghei* WT oocyst on 6 days post infection, showing highly refractile pigment granules, some stationary, others presumably powered by Brownian movement.

Title: Supplementary Movie 2 - Time lapse of *P. berghei crtl* $^{-/-}$ oocyst 6 days post infection.

Description: A representative *P. berghei crtl* $^{-/-}$ oocyst 6 days post infection with refractile pigment granules moving within enlarged vacuoles.

Title: Supplementary Movie 3 - Time lapse of *P. berghei* WT oocyst 8 days post infection.

Description: As Movie 1 but recorded on day 8 post infection. The refractile pigment granules are more stationary after commencement of sporogony.

Title: Supplementary Movie 4 - Time lapse of *P. berghei crtl* $^{-/-}$ oocyst 6 days post infection.

Description: Oocyst with Brownian movement of refractile pigment granules in larger vacuoles on day 8 post infection.
