# Supplementary material for: A CRISPR homing screen finds a chloroquine resistance transporter-like protein of the Plasmodium oocyst essential for mosquito transmission of malaria
Source: Nat Commun. 2025 Apr 24;16:3895. doi: 10.1038/s41467-025-59099-1 (PMC12022033; doi:10.1038/s41467-025-59099-1)
Supplement: Supplementary file 8 — Reporting summary [file 41467_2025_59099_MOESM8_ESM.pdf]

nature portfolio

Corresponding author(s): Oliver Billker

Last updated by author(s): April 03, 2025

Reporting Summary

Nature Portfolio wishes to improve the reproducibility of the work that we publish. This form provides structure for consistency and transparency in reporting. For further information on Nature Portfolio policies, see our Editorial Policies and the Editorial Policy Checklist.

Statistics

For all statistical analyses, confirm that the following items are present in the figure legend, table legend, main text, or Methods section.

n/a	Confirmed	
☐	☒	The exact sample size (*n*) for each experimental group/condition, given as a discrete number and unit of measurement
☐	☒	A statement on whether measurements were taken from distinct samples or whether the same sample was measured repeatedly
☐	☒	The statistical test(s) used AND whether they are one- or two-sided<br>*Only common tests should be described solely by name; describe more complex techniques in the Methods section.*
☒	☐	A description of all covariates tested
☒	☐	A description of any assumptions or corrections, such as tests of normality and adjustment for multiple comparisons
☐	☒	A full description of the statistical parameters including central tendency (e.g. means) or other basic estimates (e.g. regression coefficient) AND variation (e.g. standard deviation) or associated estimates of uncertainty (e.g. confidence intervals)
☐	☒	For null hypothesis testing, the test statistic (e.g. *F*, *t*, *r*) with confidence intervals, effect sizes, degrees of freedom and *P* value noted<br>*Give P values as exact values whenever suitable.*
☒	☐	For Bayesian analysis, information on the choice of priors and Markov chain Monte Carlo settings
☒	☐	For hierarchical and complex designs, identification of the appropriate level for tests and full reporting of outcomes
☐	☒	Estimates of effect sizes (e.g. Cohen's *d*, Pearson's *r*), indicating how they were calculated

Our web collection on statistics for biologists contains articles on many of the points above.

Software and code

Policy information about availability of computer code

Data collection	Talos L120C, Zeiss zen lite viewer, Las X (Leica), ImageJ 2.14, BD FACS Aria III.
Data analysis	FlowJo Version 10, ImageJ 2.14, Python 3.9.0, Pandas 2.2, Benchling. Cartoons in Figures 1, 2b, 3a, and 4 were drawn using Affinity Designer

For manuscripts utilizing custom algorithms or software that are central to the research but not yet described in published literature, software must be made available to editors and reviewers. We strongly encourage code deposition in a community repository (e.g. GitHub). See the Nature Portfolio guidelines for submitting code & software for further information.

Data

Policy information about availability of data

All manuscripts must include a data availability statement. This statement should provide the following information, where applicable:

- Accession codes, unique identifiers, or web links for publicly available datasets
- A description of any restrictions on data availability
- For clinical datasets or third party data, please ensure that the statement adheres to our policy

The raw data supporting the findings of this paper are available in the article (Supplementary tables). The raw barcode sequence counts and their analysis have been deposited in github. (https://github.com/vpandey-om/HomingCas9)

Research involving human participants, their data, or biological material

Policy information about studies with human participants or human data. See also policy information about sex, gender (identity/presentation), and sexual orientation and race, ethnicity and racism.

Reporting on sex and gender	Not applicable
Reporting on race, ethnicity, or other socially relevant groupings	Not applicable
Population characteristics	Not applicable
Recruitment	Not applicable
Ethics oversight	Not applicable

Note that full information on the approval of the study protocol must also be provided in the manuscript.

Field-specific reporting

Please select the one below that is the best fit for your research. If you are not sure, read the appropriate sections before making your selection.

☒ Life sciences ☐ Behavioural & social sciences ☐ Ecological, evolutionary & environmental sciences

For a reference copy of the document with all sections, see nature.com/documents/nr-reporting-summary-flat.pdf

Life sciences study design

All studies must disclose on these points even when the disclosure is negative.

Sample size	The sample size was not predetermined using statistical calculations. However, the sample sizes were adequate to perform the experiments and conduct robust statistical analyses using the specified statistical methods. The sample size (number of biological or technical replicates) is detailed in each figure legend.
Data exclusions	No data were excluded.
Replication	The number of replicates for each experiment is provided in the figure legend.
Randomization	Randomization was not applicable in this study, as there was no need for subjective judgment regarding the inclusion or exclusion of data.
Blinding	For the proof-of-principle experiments and phenotypic analysis involving microscopic readouts of oocyst colour, only one author was aware of the sample identities. The samples were numbered instead of named to ensure blinding for the other authors who collected images and analyzed the results. The samples for barcode sequencing, FACS, and Western blotting were not blinded to the authors since all measurements were objective.

Reporting for specific materials, systems and methods

We require information from authors about some types of materials, experimental systems and methods used in many studies. Here, indicate whether each material, system or method listed is relevant to your study. If you are not sure if a list item applies to your research, read the appropriate section before selecting a response.

Materials & experimental systems

n/a	Involved in the study	
☐	☒	Antibodies
☐	☒	Eukaryotic cell lines
☒	☐	Palaeontology and archaeology
☐	☒	Animals and other organisms
☒	☐	Clinical data
☒	☐	Dual use research of concern
☒	☐	Plants

Methods

n/a	Involved in the study	
☒	☐	ChIP-seq
☐	☒	Flow cytometry
☒	☐	MRI-based neuroimaging

Antibodies

Antibodies used	goat anti-FLAG (Cell Signaling Technologies, D6W5B, #14793)<br>rabbit anti-HA (3724S, Cell Signaling Technologies)<br>mouse anti alpha tubulin (00020911-1VL, Sigma)<br>Cy3 13.1 mouse monoclonal anti-P28<br>anti-mouse Alexa Fluor 647 (Thermofischer, A-32733)<br>anti-rabbit Alexa Fluor 488 (Thermofischer, A-11073)
Validation	Commercial secondary antibodies were not verified by us. Sigma's tubulin mab is the TAT-1 antibody raised by Keith Gull's lab and used for decades in Plasmodium research (e.g. Billker et al., 2002, PMID: 12503686) . For the 13.1 mab see ref. PMID: 2453831.

Eukaryotic cell lines

Policy information about cell lines and Sex and Gender in Research

Cell line source(s)	P. berghei ANKA lines used in this study are the wild type reference clone cl15cy1, the Pb Bergreen line, which expresses GFP from a silent intergenic locus on chromosome 6 under the control of the hsp70 promoter and the Pb Bern mCherry line, which uses the same promoter to express mCherry from the p230p locus (PMID: 22705315,PMID: 25786000). PV1-mCherry line has PV1 c-terminally tagged with mCherry and 3X MYC (PMID: 30232409).<br>md1- cas9, fd1-cas9, fd1-cas9 myoA gfp, md1-cas9 myoA gfp, fd1-cas9 myoA mcherry, md1-cas9 myoA mcherry, d1-cas9 myoA gfp with gRNA, md1-cas9 myoA gfp with gRNA, crtl-, crtl-HA lines were generated in this study (see methods section)
Authentication	The lines generated in this study were not authenticated by sequencing but verified by genotyping PCR, FACS andwestern blot as appropriate.
Mycoplasma contamination	Mice used for this study were specific pathogen free (including mycoplasma) and subjected to regular pathogen screening.
Commonly misidentified lines<br>(See ICLAC register)	NA

Animals and other research organisms

Policy information about studies involving animals; ARRIVE guidelines recommended for reporting animal research, and Sex and Gender in Research

Laboratory animals	Female BALB/c mice were 6 weeks old, and female Wistar Han IGS rats were 150 g upon purchase from Charles River Europe. Mice were group-housed as four cage companions, and rats as two cage companions, in individually ventilated cages with autoclaved chow, sterile drinking water, and sterile wood chip bedding and paper towels for nesting. Environmental conditions were maintained at 21°C, and 55% relative humidity under a 12/12 h light-dark cycle.
Wild animals	Not applicable
Reporting on sex	Not applicable
Field-collected samples	Not applicable
Ethics oversight	All procedures were performed according to the guidelines and approved protocols from the Umeå Centre for Comparative Biology (UCCB) at Umeå University under Ethics Permit A13-2019 and approved by the Swedish Board of Agriculture.

Note that full information on the approval of the study protocol must also be provided in the manuscript.

Plants

Seed stocks	NA
Novel plant genotypes	NA
Authentication	NA

Flow Cytometry

Plots

Confirm that:

- [ ] The axis labels state the marker and fluorochrome used (e.g. CD4-FITC).
- [ ] The axis scales are clearly visible. Include numbers along axes only for bottom left plot of group (a 'group' is an analysis of identical markers).
- [ ] All plots are contour plots with outliers or pseudocolor plots.
- [ ] A numerical value for number of cells or percentage (with statistics) is provided.

Methodology

Sample preparation	No FACS data are shown in the manuscript and the tick-boxes in this section are not applicable. FACS was used for parasite cloning and the success of cloning was verified by genotyping for absence of wild type as described. For cloning, blood was collected when parasitemia reached 0.1% to minimise double-infected red blood cells. Blood (10 µl each) was collected in 200 µl of CLB buffer (PBS, 20 mM HEPES, 20 mM Glucose, 4 mM sodium bicarbonate, 1 mM EDTA, 0.1% w/v bovine serum albumin, pH 7.25).
Instrument	BD FACS Aria III
Software	FlowJo Version 10
Cell population abundance	Initial gating was done using 100,000 events and 10 or 100 parasites were sorted for cloning.
Gating strategy	Erythrocytes were selected by gating on forward/side-light scatter, and BFP-expressing parasites were gated in the BV421A channel. Gating strategies

- [ ] Tick this box to confirm that a figure exemplifying the gating strategy is provided in the Supplementary Information.
